# Supplementary material for: Local noise in a diffusive conductor
Source: Sci Rep. 2016 Jul 28;6:30621. doi: 10.1038/srep30621 (PMC4964630; doi:10.1038/srep30621)
Supplement: Supplementary Information [file srep30621-s1.pdf]

Local noise in a diffusive conductor. Supplemental Material.

E.S. Tikhonov, D.V. Shovkun, and V.S. Khrapai
Moscow Institute of Physics and Technology, Dolgoprudny, 141700 Russian Federation and
Institute of Solid State Physics, Russian Academy of Sciences, 142432 Chernogolovka, Russian Federation

D. Ercolani, F. Rossella, M. Rocci, L. Sorba, and S. Roddaro
NEST, Istituto Nanoscienze – CNR and Scuola Normale Superiore, Piazza S. Silvestro 12, I-56127 Pisa, Italy

SCATTERING TIMES IN NANOWIRES

The shot noise measurements of Fig. 2 of the main paper demonstrate a close to universal 1/3 value of the Fano factor, indicating that our InAs nanowires (NWs) are in the regime of elastic diffusive transport. This behavior persists for bias currents up to $1\,\mu$A and bias voltages across the NW up to 10-20 mV. Corresponding quasiparticle energies measured in respect to the Fermi energy are about $\varepsilon \sim 10\,$meV. In the following, we compare this experimental observation with the ab-initio estimates of the elastic vs inelastic scattering times.

Disorder scattering

InAs NWs used in this work have a typical diameter of $d \approx 70\,$nm (hexagonal cross-section, measured from corner to corner) and length between the contacts of $L \approx 2\,\mu$m. The carrier density is about $n \approx 10^{18}\,\mathrm{cm}^{-3}$, that corresponds to a Fermi energy of $E_F \approx 160\,$meV and the Fermi velocity at $v_F \sim 1.6 \times 10^6\,$m/s. At liquid He temperatures the NW resistance is in the range $R \approx 10-20\,\mathrm{k}\Omega$, hence a resistivity of $\rho \sim \pi \mathrm{d}^2\mathrm{R}/4\mathrm{L} \approx 3\,\mathrm{m}\Omega\cdot\mathrm{cm}$. This corresponds to a mean-free path for elastic disorder scattering $l_{mfp} = 3\pi^2\rho^{-1}k_F^{-2}\hbar/e^2 \sim 40\,$nm and mobility of about $2\times 10^3\,\mathrm{cm}^2/\mathrm{Vs}$. We evaluate the quasiparticle dwell time in a NW at $\tau_{dwell} = L^2/v_F l_{mfp} \sim 60\,$ps.

Electron-electron scattering

We evaluate the electron-electron (e-e) scattering time based on the calculation of the quasiparticle life time in a 3D Fermi-liquid[1]. With the Thomas-Fermi screening wave-vector defined as $q_{TF} = (4e^2k_F^2/\pi\hbar v_F)^{1/2}$ we obtain for the e-e scattering time: $\tau_{ee} = (\varepsilon/E_F)^{-2}(e^2/32/\hbar/q_{TF}k_F^2\pi)^{-1} \propto \varepsilon^2$. With $\varepsilon = 2\,$meV this estimate results in $\tau_{ee} \sim 130\,$ps.

Electron-phonon scattering

Following Ref.[2] we express the energy relaxation rate per unit volume for deformation potential scattering on acoustic phonons in the Bloch-Grüneisen regime as $dE/dt = 6D^2m^2(k_BT_e)^5(\pi^3\rho_m\hbar^7s^4)^{-1}$. Here k_B is the Boltzman constant, T_e is the electronic temperature, $D = 4.9\,$eV, $s \approx 4.3\,$km/s, $\rho_m \approx 5.7\times 10^3\,\mathrm{kg/m}^3$ and $m \approx 0.023\,m_e$ are, respectively, the deformation potential, typical sound velocity, mass density and the effective mass of the electrons in InAs. Here we assumed that the bath temperature is small compared to T_e. The electron-phonon energy relaxation rate can be estimated as[3] $\tau_{e-ph}^{-1} = (dE/dt)(C_eT_e)^{-1}$, where $C_e = (\pi/3)^{2/3}m\hbar^{-2}n^{1/3}(k_B)^2T_e$ is the electronic heat capacitance per unit volume. For a typical excess energy of $\varepsilon = k_BT_e$ we obtain $\tau_{e-ph}^{-1} = (dE/dt)(\pi/3)^{-2/3}\hbar^2m^{-1}n^{-1/3}\varepsilon^{-2} \propto \varepsilon^3$. With $\varepsilon = 2\,$meV this results in $\tau_{e-ph} \sim 30\,$ps.

While the above estimates are consistent with the elastic diffusive transport in our InAs NWs for quasiparticle energies up to $\varepsilon = 2\,$meV, one would expect the e-ph energy relaxation to come into play at higher excitations. This would manifest itself in a reduction of the shot noise spectral density from the universal Fano factor value $F = 1/3$ already around $I \approx \varepsilon/eR \sim 150\,$nA, which is not observed in our experiment, see Fig.2 of the main paper. Such an apparent weakening of the e-ph interaction in InAs NWs may result from screening of the e-ph interaction and/or reduced quasi-1D phase space for scattering. On the other hand, we can not exclude the weak e-ph energy relaxation originates from a bottle neck process of escape of the non-equilibrium phonons from the NW. In this case, the effective lattice temperature would follow that of the electronic system, and one could neglect the shot noise suppression. Still, the experimental $F \approx 1/3$ would be a pure coincidence in such a situation.

SCATTERING LENGTH SCALES IN THE CONTACT METAL

Similarly, we estimated the length scales relevant for the metallic constriction, consisting predominantly of Au. Here we used $n \approx 7\times 10^{22}\,\mathrm{cm}^{-3}$, $m \approx 1.1\,m_e$, $E_F = 5.6\,$eV, $D = 2E_F/3$, $s \approx 3.2\,$km/s, $\rho \approx 3.2\,\mu\Omega\cdot$cm and $\rho_m \approx 19.3\times 10^3\,\mathrm{kg/m}^3$. With these parameters, the dwell time in the constriction ($L \approx 2\,\mu$m) is estimated at $\tau_{dwell} \sim 130\,$ps, while the inelastic time scales for a quasiparticle energy of $\varepsilon = 1\,$meV are $\tau_{ee} \sim 16\,$ns and $\tau_{e-ph} \sim 400\,$ps. This estimates correspond to a local thermalization length via e-e scattering of about $\sim 25\,\mu$m and the energy relaxation length via e-ph interaction of about $\sim 3.5\,\mu$m, both longer than L. Note, however, that while the inefficiency of the e-ph relaxation follows directly from the linear dependencies of T_S on I_H in Figs. 4 and 5 of the main paper, the role of the e-e processes remains hidden in our experiments. For example, even a

tiny concentration of magnetic impurities might strongly accelerate thermalization via e-e processes, see e.g.[4–6].

That $\tau_{dwell} < \tau_{ee}\,, \tau_{e-ph}$ is consistent with the observation of linear dependence of the measured noise temperature on the driving current in Fig. 4 of the main article. Regarding e-ph interaction, the above parameters correspond to the e-ph energy relaxation rate of $\Sigma \approx 0.1\,\mathrm{nW}\mu\mathrm{m}^{-3}\mathrm{K}^{-5}$, which is close to a free-electron model estimate[7] and a factor of 5 smaller than the values used in the literature[8,9]. Possible discrepancy can be regarded minor in view of the strong energy dependence of τ_{e-ph}.

[1] J. J. Quinn and R. A. Ferrell, Phys. Rev. **112**, 812 (1958).
[2] B. K. Ridley, Reports on Progress in Physics **54**, 169 (1991).
[3] P. W. Anderson, E. Abrahams, and T. V. Ramakrishnan, Phys. Rev. Lett. **43**, 718 (1979).
[4] A. Kaminski and L. I. Glazman, Phys. Rev. Lett. **86**, 2400 (2001).
[5] G. Göppert and H. Grabert, Phys. Rev. B **64**, 033301 (2001).
[6] A. Anthore, F. Pierre, H. Pothier, and D. Esteve, Phys. Rev. Lett. **90**, 076806 (2003).
[7] M. Kaganov, I. Lifshitz, and L. Tanatarov, JETP **4**, 173 (1957).
[8] A. H. Steinbach, J. M. Martinis, and M. H. Devoret, Phys. Rev. Lett. **76**, 3806 (1996).
[9] M. Henny, S. Oberholzer, C. Strunk, and C. Schönenberger, Phys. Rev. B **59**, 2871 (1999).
